# Supplementary material for: The association between the rise of gun violence in popular US primetime television dramas and homicides attributable to firearms, 2000–2018
Source: PLoS One. 2021 Mar 17;16(3):e0247780. doi: 10.1371/journal.pone.0247780 (PMC7968679; doi:10.1371/journal.pone.0247780)
Supplement: S1 Appendix — (DOCX) [file pone.0247780.s001.docx]

**S1 Appendix**

US prime-time TV-14 dramas in the sample by number of segments, years coded, and peak rank as listed in Variety Magazine, 2000-2018

| TV show title | Genres | Mean Segments | Years Coded | Peak Rank |
| --- | --- | --- | --- | --- |
| Blindspot | Police | 54 | 2 | 21 |
| Blue Bloods | Police/Legal | 88.4 | 9 | 7 |
| Bones | Police/Medical | 81 | 4 | 26 |
| Bull | Legal | 60 | 3 | 4 |
| Chicago Med | Medical | 69.5 | 4 | 19 |
| Code Black | Medical | 51 | 3 | 26 |
| Criminal Minds | Police | 97 | 14 | 10 |
| Crossing Jordan | Police/Medical | 54.5 | 2 | 20 |
| CSI: Crime Scene Investigation | Police | 98 | 15 | 1 |
| CSI: Miami | Police | 97.7 | 10 | 5 |
| CSI: NY | Police | 81 | 9 | 15 |
| Elementary | Police | 72.3 | 3 | 15 |
| ER | Medical | 80.4 | 10 | 2 |
| Grey's Anatomy | Medical | 86.6 | 13 | 5 |
| Hawaii Five-0 | Police | 87 | 9 | 12 |
| House M.D. | Medical | 86.3 | 7 | 7 |
| How to Get Away with Murder | Police | 36 | 2 | 19 |
| JAG | Legal | 81 | 4 | 14 |
| Law & Order: Criminal Intent | Police/Legal | 82.8 | 5 | 19 |
| Law and Order | Police/Legal | 54 | 2 | 12 |
| Law and Order: Special Victims Unit | Police/Legal | 93.6 | 10 | 12 |
| NCIS | Police/Medical | 100.5 | 16 | 1 |
| NCIS: Los Angeles | Police/Medical | 97.2 | 10 | 4 |
| NCIS: New Orleans | Police/Medical | 86.2 | 5 | 4 |
| Private Practice | Medical | 54 | 2 | 29 |
| The Blacklist | Police | 61.4 | 5 | 6 |
| The Good Wife | Legal | 89 | 8 | 14 |
| The Mentalist | Police | 86.6 | 8 | 6 |
| Unforgettable | Police | 49.5 | 2 | 24 |
| Without A Trace | Police | 77.3 | 7 | 6 |
| Mean |  | 83.9 | 7.0 |  |
